# Dr. John W. Robinson

**Published:** 1900-11

**Authors:** 


### OBITUARY NOTES.

Dr. John W. Robinson, of Lyons, N.Y., died suddenly of some affection of the heart, October 21, 1900, aged 57 years. He was a graduate of the College of Physicians and Surgeons, New York, Class of '67.—Dr. J. L. Cutler, of Boliver, N.Y., died at his home October 14, 1900, aged 71 years. He was a graduate of the University of New York, Class of '50. He held many local offices, among which was examiner for pensions during eight years.—Dr. Charles St. John of the Class of '98, who went to the Philippines as an acting assistant surgeon, U. S. Army, is reported to have died near Manila of typhoid fever early in October.—Dr. Charles Roemmelt, of Elmira, U. of B., '98, died July 20, 1900, while serving as acting assistant surgeon, U. S. Army, in the Philippines.—Dr. Samuel Smith Purple, one of the older and most respected physicians of New York City, died at his home September 29, 1900. He presented to the New York Academy of Medicine some years ago 4,000 volumes, which formed the nucleus of its present splendid library. Dr. Purple retired from active practice about fifteen years ago.

